# Endocrine responses of the stress system to different types of exercise

**DOI:** 10.1007/s11154-022-09758-1

**Published:** 2022-10-15

**Authors:** Nikolaos Athanasiou, Gregory C. Bogdanis, George Mastorakos

**Affiliations:** 1 Unit of Endocrinology, Diabetes mellitus and Metabolism, School of medicine, ARETAIEION hospital, National and Kapodistrian University of Athens, Neofytou Vamva str 10674, Athens, Greece; 2 Dermatology Department, Evangelismos General hospital, Athens, Greece Ipsilantou 45-47, 10676; 3 School of Physical Education and Sports Science, National and Kapodistrian University of Athens, 17237 Dafne, Greece

**Keywords:** Exercise, Cortisol, HPA, Catecholamines, IL-6, GH

## Abstract

Physical activity is an important part of human lifestyle although a large percentage of the population remains sedentary. Exercise represents a stress paradigm in which many regulatory endocrine systems are involved to achieve homeostasis. These endocrine adaptive responses may be either beneficial or harmful in case they exceed a certain threshold. The aim of this review is to examine the adaptive endocrine responses of hypothalamic-pituitary-adrenal axis (HPA), catecholamines, cytokines, growth hormone (GH) and prolactin (PRL) to a single bout or regular exercise of three distinct types of exercise, namely endurance, high-intensity interval (HIIE) and resistance exercise. In summary, a single bout of endurance exercise induces cortisol increase, while regular endurance exercise-induced activation of the HPA axis results to relatively increased basal cortisolemia; single bout or regular exercise induce similar GH peak responses; regular HIIE training lowers basal cortisol concentrations, while catecholamine response is reduced in regular HIIE compared with a single bout of HIIE. HPA axis response to resistance exercise depends on the intensity and volume of the exercise. A single bout of resistance exercise is characterized by mild HPA axis stimulation while regular resistance training in elderly results in attenuated inflammatory response and decreased resting cytokine concentrations. In conclusion, it is important to consider which type of exercise and what threshold is suitable for different target groups of exercising people. This approach intends to suggest types of exercise appropriate for different target groups in health and disease and subsequently to introduce them as medical prescription models.

## Introduction

Living organisms survive by maintaining a complex dynamic equilibrium called homeostasis, which is constantly challenged by intrinsic or extrinsic disturbing factors, called *stressors* [1]. The homeostasis of the organism can be threatened by external or internal stressors. When a threshold of the described complex dynamic equilibrium is reached, an adaptive compensatory response (called *stress response*), is initiated as part of an innate process developed through evolution to protect the organism *via* re-equilibration of the homeostasis (called *allostasis*). If the adaptive response exceeds a certain level it can become harmful to the organism [2].

### Exercise and the stress system

The stress response is mediated by the *stress system*, which comprises central and peripheral components. The main central components of the stress system are the hypothalamic-corticotroph (pituitary) axis, with the hypothalamic corticotropin-releasing hormone (CRH) and the arginine vasopressin (AVP) neurons as main protagonists, and the locus caeruleus (LC) located in the pons [i.e. the LC/norepinephrine (NE) system] which secretes predominantly NE [3]. Regarding the hypothalamic-corticotroph axis, during stress response, CRH and AVP are synthesized in the hypothalamic paraventricular nuclei, are secreted into the hypophyseal portal system, reaching the anterior pituitary lobe where they lead to the release of adrenocorticotropic hormone (ACTH) into the systemic circulation [3]. Peripheral actions of the stress response include those from the adrenal effectors of the hypothalamic-pituitary-adrenal (HPA) axis, the sympathetic and the parasympathetic system [4]. ACTH principally promotes the synthesis of glucocorticoids by the adrenal zona fasciculata, while it also regulates adrenal androgen secretion by the zona reticularis and aldosterone secretion by the zona glomerulosa. Glucocorticoids exert a negative feedback loop at the hypothalamic and the pituitary level *via* the CRH neuron and the eosinophilic ACTH-producing corticotroph cell, respectively [5]. Under stress, locus ceruleus activates α1-adrenoceptors on sympathetic pre-ganglionic neurons in the spinal cord increasing thus, sympathetic activity. Furthermore, it activates α2-adrenoreceptors on pre-ganglionic parasympathetic neurons reducing thus, parasympathetic activity. Subsequently, in prolonged stress the sympathetic system is continuously activated in the absence of a parasympathetic counter-activity (predominantly in the upper gastrointestinal system), while the large bowel is stimulated by the sacral parasympathetic system [1]. The central functions of the stress response include arousal, aggression, alertness, as well as inhibition of vegetative functions such as reproduction, feeding and growth. The main peripheral functions include increase of, respiration, cardiovascular tone, oxygenation, metabolism and energy substrate availability.

As we have suggested in the past, exercise is a stress situation in which most systems of the human body (i.e. cardiovascular, respiratory, immune, endocrine, musculoskeletal) are involved and *via* adaptation they attain allostasis [3]. During exercise, homeostasis is controlled by many regulatory hormonal systems including the HPA axis and the the LC/NE system, hormones such as prolactin (PRL) and growth hormone (GH), while the cytokines system is also involved [6]. Regular exercise modifies the homeostatic capacity of most systems of the human body *via* long-lasting adaptations of their function. These adaptations can be beneficial for adult and young individuals, and include improvement of cardiorespiratory fitness and muscle strength. Regular exercise decreases the risk for cardiovascular disease and type 2 diabetes mellitus, as well as the risk for some forms of cancer [7].

The minimum weekly volume of physical activity necessary for the induction of positive effects upon health is 150 min of moderate-intensity endurance exercise or 60–75 min of vigorous activity, or a combination of both [8]. In addition to endurance exercise, two sessions of resistance training per week have been proposed as a necessary form of complementary training, in order to preserve and increase muscle strength and mass [8]. In the last 15 years, high intensity interval exercise (HIIE) has also been employed as an effective and time-efficient exercise modality for healthy and diseased individuals [9]. During this type of exercise, a considerable part of the energy is derived through oxygen-dependent metabolism, while phosphocreatine degradation and glycolysis are greatly activated [10]^,^[11]. The adaptations caused by the short duration HIIE protocols (20 min) are similar to those induced by much longer duration moderate intensity exercise, and thus they are often used as time-efficient, despite the fact that they are much more demanding [9]^,^[12]^,^[13]. All types of exercise represent physical stressors which challenge the homeostasis of different systems. This challenge can happen once following a single bout of exercise (called by some authors acute exercise) or in a regular/routine/long-standing/recurrent manner (called by some authors chronic exercise) [3]. The stress responses to exercise may vary greatly, depending on intensity (high-moderate-low), duration (short-moderate-long) and type of exercise (continuous vs. intermittent), leading to different levels of allostasis which may also be influenced by other parameters such as age, sex and training status. The aim of the present critical review is to examine the adaptive endocrine responses of the human stress system to a single bout of exercise or to regular exercise of three distinct types of exercise, namely endurance exercise, HIIE and resistance exercise.

### Types of exercise

#### Endurance exercise

Endurance exercise is the most studied type of exercise. Endurance exercise is any exercise which involves large muscle groups, and relies mainly on oxygen-dependent metabolism [14]. Its duration varies from a few minutes to several hours and is related inversely to exercise intensity, while trained individuals can maintain higher intensity for longer time periods. For this type of exercise, performance depends on maximal oxygen uptake (VO_2max_), blood lactate threshold (i.e. the exercise intensity at which the blood concentration of lactate begins to exponentially increase [15]), and energy efficiency or the oxygen cost for performing exercise against a certain external load [16]. In this case, the term “lactate threshold” is employed to showcase lactate as a gluconeogenic precursor without implying any relationship to “anaerobiosis” or tissue oxygenation [17]. Energy metabolism during endurance exercise relies on fat and carbohydrate oxidation, with the latter contributing predominantly when intensity is higher [18]. Apart from the improvement of cardiorespiratory function, adaptations to endurance training include upregulation of pro- and anti- oxidation, mitochondrial biogenesis and angiogenesis [6].

#### High intensity interval exercise (HIIE)

HIIE has been proposed as an alternative way of combined endurance and high intensity exercise, which is less time-consuming and induces similar adaptations to prolonged endurance exercise[9]^,^[12]^,^[13]. HIIE refers to short duration bouts of exercise performed at high intensity (typically above 85% VO_2max_), repeated for several times after a specific recovery interval[19]. It can be classified into two distinct exercise formats:

- high-intensity interval exercise (HIIE), performed at intensities between 85% and 100% VO_2max_, with bout duration from 12 s to 4 min (1 min being more common)[20]^,^[12], and equal recovery intervals, and.
- sprint interval exercise (SIE), performed all-out (corresponding to intensities > 150–200% VO_2max_), with bout duration from 5 to 30 Sect. [11]

Of note, phosphocreatine degradation and glycolysis contribute largely to energy supply in HIIE (phosphocreatine degradation and glycolysis) during the initial bouts of exercise, especially during SIE (> 80% of the energy is of glycolytic origin in the first bouts) [21]. During repeated bouts of intense exercise, blood and muscle lactate concentrations reach their peak, while oxygen-dependent metabolism contributes increasingly more to the total energy supply [19]. Consequently, both SIE and HIIE largely activate oxygen-dependent metabolism and are considered effective and time-efficient methods for improving physical performance and health [22].

#### Resistance exercise

Resistance exercise involves training against an external load, that may be in the form of weights, machines or other means, such as elastic bands [23]. The intensity of resistance exercise is determined by the load that can be lifted or moved only once and is called one-repetition maximum (1RM). Depending on the %1RM used and the number or repetitions, a resistance training program may be characterized as targeting muscle hypertrophy (typically 65–85%1RM, with rest intervals of 30–90 s), muscle strength (> 85%1RM, with 2–3 min rest) or muscle endurance (< 65%1RM with short resting intervals) [23]. However, recent evidence suggests that muscle hypertrophy can be achieved by using much lower loads (i.e. 30% 1RM), provided that repetitions are performed to exhaustion [24].

## Endocrine responses of the stress system to a single bout of endurance exercise or to regular endurance exercise

### HPA axis

A single bout endurance exercise stimulates HPA axis. Stress-induced CRH stimulates pro-opiomelanocortin (POMC) in the pituitary, as reflected by the increase in the periphery, of POMC-derived peptides such as β-endorphin and β-lipotropin [3]. β-Εndorphin inhibits the stress system as well as the hypothalamic-pituitary-gonadal axis, while it promotes analgesia *via* inhibition of ascending stimuli to the hindbrain and the spinal cord [4]. Exercise intensity and duration determine the HPA axis response to endurance exercise [25]. The minimum intensity of a single bout of endurance exercise (i.e. the “threshold”) necessary to produce an HPA axis-induced cortisol response corresponds to 60% of maximum oxygen uptake (VO_2max_). Above 60% VO_2max_, a linear correlation between the intensity of exercise and the increase in plasma ACTH and cortisol concentrations is observed [26]. If exercise intensity is below this threshold, HPA axis might be activated only after prolongation of the exercise duration (i.e. 90 min of exercise with at least 40% VO_2max_) [26]. Cortisol concentrations return to pre-exercise values in about 150 min after the end of a single bout of intensive or prolonged endurance exercise [25]. The recovery time between bouts of endurance exercise modulates the intensity of the HPA response. In male elite endurance athletes, a single repeat of a prolonged strenuous endurance exercise bout (e.g. 75 min of exercise at 75% VO_2max_) induces a more pronounced increase in ACTH and cortisol when a previous bout of endurance exercise is performed 3 h (compared to 6 h) earlier. Even though plasma concentrations of cortisol and ACTH normalize between bouts of endurance exercise, HPA axis activity is enhanced when exercise is repeated after a shorter recovery period [25]. Of note, glucocorticoids increase the availability of metabolic substrates for muscles, while they regulate vascular function and responsiveness during exercise. Furthermore, glucocorticoids decrease the severity of exercise-induced muscle damage by controlling the immune/inflammatory reaction [27].

On the other hand, basal evening ACTH and cortisol concentrations in highly trained athletes are found elevated compared with sedentary subjects and moderately trained runners [28]. Thus, regular exercise-induced activation of the HPA axis results to a mild basal hypercortisolemia, particularly in highly trained endurance runners (Table 1). ACTH hypersecretion and ensuing adrenal enlargement has been reported after regular daily strenuous endurance exercise [29]. Interestingly, in these trained runners, cortisol response following a single bout of endurance exercise, is lower than concentrations one would expect in this exercise condition, suggesting the development of adaptive mechanisms to the chronically repeated stressor of exercise.

**Table 1:** Basal and post-exercise concentrations of cortisol, catecholamines, cytokines and GH in different types of exercise. ↑= mild increase; ↑↑= moderate increase; ↑↑↑=intense increase; ↔=same as in untrained individuals; ?=unclear; NE = Norepinephrine; E = Epinephrine; GH = Growth hormone; IL-6 = Interleukin 6; TNF = Tumor necrosis factor; Il-1 = Interleukin 1; HIIE = high intensity interval exercise. The cross (+) corresponds to obese individuals; the asterisk (*) corresponds to young individuals; the pound sign (#) corresponds to elderly individuals

Type of exercise			Cortisol		Catecholamines (E, NE)	Cytokines (TNF, IL-1. IL-6)		GH
**Single-bout endurance**		Post-exercise		↑↑	↑↑		↑↑TNF ↑↑IL-1 ↑↑IL-6	↑↑
**Regular** **endurance**		Basal		↑	**↔**?		↑ IL-6	**↔** ?
		Post-exercise		↑	↑↑↑		↑ IL-6 ↑TNF	↑↑
**Single-bout HIIE**	Post-exercise			↑↑ ↓^+^	↑↑		↑↑TNF ↑↑IL-6	↑↑
**Regular HIIE**		Basal		↓	**↔**?		↑↑IL-6	?
		Post-exercise		↑	↑		?	?
**Single-bout resistance**	Post-exercise			↑	↑↑		↑↑TNF ↑ IL-6* ↑↑ IL-6^#^	↑↑
**Regular** **resistance**		Basal		↓	?		↑ IL-6* ↑↑ IL-6^#^	**↔**
		Post-exercise		↑	?		↑IL-6	↑↑

### Catecholamines

The sympathetic system and its efferent organ, the adrenal medulla (catecholaminergic), participate in the “fight or flight” response to a stressor. Sympathetic nervous system and plasma catecholamine concentrations regulate hepatic glucose production and lipid metabolism during endurance exercise [29].

An increase in norepinephrine levels is seen within 15 min of a single bout of endurance exercise before any changes in epinephrine concentrations are noticed [30]. Norepinephrine stimulates directly hepatic gluconeogenesis *via* activation of β-adrenergic receptors in hepatocytes [31]. The need of substrate mobilization is increased in prolonged exercise and this is attained by exercise-induced epinephrine secretion which promotes lipolysis [32]. The catecholamine response to endurance exercise depends on both intensity and duration. Regardless of intensity, secretion of catecholamines increases continuously until exhaustion [29]. Of note, the minimum necessary duration of a single bout endurance exercise for ignition of catecholamines secretion is still unknown, although in many types of exercise and especially in competition, the increase of catecholamines anticipates the beginning of exercise. Norepinephrine concentrations increase as a function of exercise intensity when exercise duration remains unchanged [29]. Of note, secretion of catecholamines is increased overproportionally above a certain level of lactate concentration [33]. Post-exercise epinephrine and IL-6 concentrations are greater in the evening than in the morning suggesting that an evening single bout of endurance exercise may trigger greater exercise-induced lipolysis compared with a morning single bout of exercise. Of note, exercise-associated lipolysis depends also on the action of other lipolytic hormones such as GH or glucocorticoids (the former more elevated around midnight and the latter around 08.00 am in the morning) as well as on the type, duration and intensity of the exercise [34]. Also, epinephrine and norepinephrine concentrations are greater when exercise requires upper body involvement (arms) rather than lower body (legs) at similar VO_2max_ values [35].

Endurance trained athletes demonstrate greater epinephrine response during a single bout of endurance exercise compared to untrained controls exercising at the same relative intensity. This chronic adaptation is called “sports adrenal medulla” [29]. Interestingly, in these endurance trained athletes the increased catecholamine response is also observed following other stress-inducing stimuli, such as hypoglycemia and hypoxia [29]. In laboratory animals, regular strenuous exercise is associated with adrenal hypertrophy and subsequently with greater total adrenal medullary epinephrine content [36].

Differences in male and female anatomy (lungs/airway size, musculature, etc.) and physiology (muscle capillarization, menstrual cycle, sex hormone concentrations) affect accordingly the responses of the two sexes to exercise. For instance, female musculature (i.e. diaphragm, a strong respiratory muscle) seems to be more resistant to exercise-elicited fatigue compared to male musculature at similar workload in high intensity endurance exercise [37]. There are only few studies that have examined the effect of estrogens on catecholamine secretion during exercise. Amenorrhoeic athletes have reduced adrenergic response to intense exercise and this could affect their performance by altering the cardiovascular and metabolic adaptations to exercise [38]. However, when these women are treated with estrogens, they show greater catecholamine secretion in response to a single bout of endurance exercise compared to women treated with *placebo* [39]. Interestingly, according to some studies, secretion of catecholamines during exercise is slightly greater during the luteal phase of menstrual cycle in comparison to the follicular phase, but further research is needed to investigate the responses of catecholamines to exercise at different phases of the menstrual cycle [40].

### Role of cytokines

The stress system is actively responding to many distinct circadian, neurosensory and blood-borne signals. These signals include cytokines such as tumor necrosis factor (TNF), interleukin 1 (IL-1), and interleukin 6 (IL-6) which participate in immune-mediated inflammatory reactions. In the past, we have shown that in humans, moderate doses of IL-6 cause an increase in plasma concentrations of ACTH and cortisol, well above the concentrations achieved with standardized doses of ovine CRH, a major stimulator of the HPA axis [41]. On the other hand, plasma IL-6 concentrations increase significantly during a single bout endurance exercise, whereas muscle damage following severe and/or prolonged endurance exercise is accompanied by cytokines secretion from macrophages, fibroblasts, and endothelial cells present in the muscles [28]^,^[42],[43]. The exercise-induced increase of IL-6 is associated to norepinephrine and epinephrine increase [42]. Of note, muscle damage is a quite different condition than muscle at exercise. According to certain authors, muscles at exercise secrete IL-6 independently of catecholamine mediation, although both are increased [44]. During exercise, IL-6 is produced mainly in muscle fibers *via* a TNF-independent pathway and is characterized as a myokine, i.e. a cytokine released by working muscles [45]^,^[46]. The subsequent activation of the HPA axis leads to glucocorticoids secretion, which in turn, inhibit ultimately the expression of inflammation-promoting cytokines and stimulate the production of inflammation-limiting cytokines. The latter regulate the function of immune cells, such as monocytes and neutrophils [47][48][49][50]. Following a single bout of exercise, monocyte chemoattractant protein 1 (MCP-1), IL-8 and IL-6 increase substantially, while IL-4, IL-10 and IL-13 increase only slightly, with similar responses in young and elderly men [51][52]. In untrained individuals, the expression of cytokines promoting inflammation 2 h post exercise is greater in the elderly than in the young men. This difference is attenuated by training, apparently due to the adaptive increase of basal cortisol secretion following systematic regular endurance exercise [51]. Thus, exercise training may contribute to attenuation of the inflammatory response in elderly people and therefore, regulate muscular regeneration and adaptation [51].

Variable responses in IL-6, TNF and vascular endothelial growth factor (VEGF) concentrations have been observed after endurance exercise training. Endurance exercise training, endurance exercise duration and physical stress may contribute to the response variability of these substances. In athletes, after prolonged endurance exercise, their concentrations seem to change to a lesser degree than in non-athletes [53]. The release of angiogenic and inflammation-promoting cytokines is increased after a single bout of endurance exercise [54]. In healthy individuals, certain authors have shown that VEGF production is acutely stimulated by endurance exercise, thus contributing to maintenance and growth of the vasculature and the mobilization of angiogenic cells [55]^,^[56]^,^[57], whereas others found unchanged or even decreased VEGF concentrations after intense or prolonged exercise [58]^,^[59]. It is suggested that VEGF increases 10 min after the onset of exercise or after completion of a single bout of exercise, and returns to resting values shortly after peaking [56]^,^[57]. These conflicting results may be explained by the different time of blood sampling in the different studies [59].

Interleukin-6 is a key mediator in the activation of the HPA axis during exercise. However, it seems that the HPA axis response to cytokines adapts to repeated bouts of IL-6. In the past we have shown that following the 7th daily consecutive administration of subcutaneous IL-6 injection to cancer patients, the initially observed exaggerated elevation of ACTH and cortisol concentration was attenuated, indicating the development of an adaptive dynamic new set-point for the stimulation of the HPA axis (centrally and peripherally) [41].

Chronic effects of exercise in relation to cytokine concentrations have been examined in trained athletes. There is evidence that young, endurance-trained athletes demonstrate increased resting IL-6 levels with attenuated inflammatory response to exercise in comparison to non-athletes, i.e. lower post-exercise IL-6 concentrations as compared to non-athletes [60]^,^[53]. In the past, we have shown that in patients suffering from sarcoidosis basal IL-6 concentrations were greater than those of healthy controls due to the chronic inflammatory state of the disease [61]. When patients with sarcoidosis performed a single bout of treadmill exercise, IL-6 concentrations increased further to concentrations greater than those of the healthy controls. However, the ACTH and cortisol concentrations of these patients, although greater than in healthy control subjects at baseline, they increased, during the same bout of treadmill exercise, only as much as the concentrations of the same hormones in healthy control subjects. It is possible that developing adaptive mechanisms (i.e. down-regulation of IL-6 receptors in the pituitary and the adrenal cortex or increased catabolism of circulating IL-6) might lead to functionally attenuated response of the HPA axis to the chronically increased IL-6 concentrations [62]. Thus, both endurance exercise training and chronically increased IL-6 blood concentrations due to disease or repeated IL-6 administration, seem to blunt the HPA axis response [41]^,^[53].

### Growth hormone and prolactin

Besides activation of the HPA axis, IL-6 is known to stimulate two other hormones: GH and PRL, which on the evolutionary scale are also related to the stress response.

GH response to endurance exercise is regulated by GH-releasing hormone (GHRH) which is secreted from the hypothalamus and acts on somatotropic cells of the anterior pituitary [63]. Different types of exercise modify the endurance exercise-induced GH response [64]. Several studies showed that GH concentrations increase with both continuous endurance and intermittent exercise with a significant increase appearing after 15 min of exercise [65, 66]. There is evidence that endurance exercise-induced GH release is achieved through the JAK-STAT5 signaling pathway [67]. Although it is suggested that there may be a linear dose-response pattern of GH secretion with increasing endurance exercise intensities, the optimal exercise intensity for GH release is still unclear [68]. Previous studies have shown that noticeable GH responses are reported at 50% VO2max, with a maximal GH stimulation at 70% VO2max and no further increase at 90% VO2max [69]. Although it has been suggested that blood lactate may trigger GH responses, there is evidence which negates an important role of endurance exercise-induced lactate on GH release. In one study, PRL and GH concentrations were measured after exogenous lactate infusion and were clearly lower than those following endurance exercise-induced increase of plasma lactate concentrations [69]. Also, an increase of GH at an intensity of 50% VO2max has been reported in the absence of lactate production [69]. Even a short-duration single bout of moderate-intensity endurance exercise (75%VO2max for 10 min) suffices to cause an exercise-induced GH response [66]. The magnitude of GH increase is approximately 300–500% above resting concentrations and can last up to 3 h post exercise [65]. Endurance exercise-induced GH response does not depend on the diurnal rhythm of GH release, as the GH response to exercise is not related to the time of day [65]^,^[70]. Interestingly, endurance exercise-induced GH release is lower than that induced by exogenous GHRH administration. Thus, during endurance exercise, the GH pathway either is not stimulated as much as following exogenous GHRH administration (which corresponds to pharmacological doses of GHRH) or that an inhibitory agent, such as somatostatin, might be involved [71]. The latter is stimulated by CRH which is found elevated during endurance exercise [3]. Furthermore, glucocorticoids inhibit GH release and antagonize GH in tissue and muscle metabolism [72].

Repeated endurance exercise bouts seem to override the GH negative feedback upon GHRH [73]. Regular endurance exercise training appears to alter GH-IGF axis considering that in amenorrheic athletes, GH half-life and volume secretion per pulse are decreased, while GH number of pulses is increased compared to that in eumenorrheic women [74]. However, after 4 months of regular endurance training in competitive cyclists, no difference was observed in exercise-induced GH secretion after a single bout of endurance exercise on a cycle ergometer, suggesting that training does not seem to affect the GH peak response to endurance exercise [75].

Interestingly, PRL response to endurance exercise was smaller than that resulting from other stimulators, such as thyrotropin-releasing hormone (TRH) or metoclopramide [69]. Of note, TRH-induced PRL stimulation is due to pharmacological doses of TRH. In the search of a release mechanism linking PRL increase and metabolic responses to endurance exercise, blood lactate and IL-6 have been studied [69]. Although lactate may play some role in PRL secretion, IL-6 seems to be far more important. A dose-response study of GH and PRL to increasing doses of recombinant human IL-6 revealed that GH release increased dramatically, while PRL showed a similar but less pronounced response [76]. Thus, it can be postulated that the increases of GH and PRL during endurance exercise are rather related to IL-6 than to lactate stimulation [3]. Of note, in the reported study, doses of administrated IL-6 resulted to concentrations of this cytokine up to 4 ng/ml in peripheral blood (corresponding to 10 mg/kg, the greatest injected dose) while during endurance exercise circulating IL-6 concentration does not usually exceed 120 pg/ml [77]. In addition, there are conflicting results regarding PRL response to regular exercise. Studies have shown that after a TRH challenge test, PRL increased more in endurance-trained athletes compared to sedentary controls [78]. Others have failed to show a difference in PRL increase following 30 min of cycling exercise between trained and sedentary male subjects [78]. The administration of a 5- hydroxytryptamine (5-HT) agonist to endurance athletes and untrained subjects resulted to a lower PRL peak concentration in comparison to untrained subjects. This result was attributed to downregulation of central 5-HT receptors following regular exercise [79]. PRL has also been suggested as a marker of overtraining syndrome but further investigation is necessary to confirm this hypothesis [80].

## Endocrine responses of the stress system to a single bout of HIIE or to regular HIIE

### HPA axis

There are only few studies on the effects of HIIE on HPA axis regulation. In a recent study 8 male volunteers completed a 3-week HIIE training with 9 HIIE sessions of 4–6 × 30 s high-intensity cycling bouts. A single bout of HIIE session induced an increase in cortisol concentration 30 min after the exercise, returning to basal levels at 24 [81].

In another study, participants performed functional HIIE in the gym at 7:00 p.m. They maintained a continuous increase of salivary cortisol from post training until 7:00 a.m., as opposed to a decline of cortisol production at 11:00 p.m., in the non-training day [82]. However, in obese individuals an acute decrease in cortisol was reported after a single HIIE bout, compared with isoenergetic resistance training or a combination of HIIE and resistance training [83]. Of note, baseline cortisol concentrations were elevated in these obese individuals compared to normal weight, healthy individuals [84].

Regular HIIE seem to decrease cortisol basal concentration. According to a study, after a 3-week HIIE training an average 42% reduction of cortisol basal concentration was observed compared to pre-training values [81].

### Catecholamines

There are few published data regarding HIIE and catecholamines. Plasma epinephrine and norepinephrine concentrations increased acutely after 10 × 6 s cycle ergometer sprints with 30 s recovery in young non-specifically trained male subjects [85]. Following high intensity exercise the elevation of epinephrine and nor-epinephrine is transient and returns to baseline in the first post-exercise hour [86].

Regular HIIE training (for 7 weeks) is associated with blunted catecholamine responses [87]. In addition, male and female adolescents and young adults might exhibit different catecholamine responses to repeated HIIE bouts. In adults, epinephrine responses were greater in men than women, but norepinephrine responses were similar. However, norepinephrine responses were greater in women than in girls, while post exercise epinephrine was greater in men than in boys [88]. It seems that menstrual phase does not influence sympatho-adrenergic responses in young untrained women [89]. Finally, 6 months of HIIE training in adolescent girls induced an increased epinephrine response, which disappeared following 5 months of detraining [90].

### Role of cytokines

The effects of a single bout of HIIE on cytokine responses are similar to those of intense endurance exercise, with an initial rise in inflammation-promoting cytokines (TNF, IL-1a, IL-6), followed by an increase in inflammation-promoting cytokines inhibitors and inflammation-limiting cytokines (IL-1 receptor antagonist, the soluble type of IL-1 receptor, the soluble TNF receptor 1 and TNF receptor 2, IL-10) [91]. In a recent study, young adults performed HIIE on a cycle ergometer at 90% VO_2max_ for 12 min (6 × 1 min bouts separated by 1 min rest intervals) and IL-1, IL-6 and TNF concentrations increased in the circulation as early as 5 min following exercise initiation [92]. In another study, where seven healthy untrained subjects performed HIIE on cycle ergometer, the main cytokine responses included an increase of IL-6 towards the end of the exercise and a decrease of MCP-1 (Monocyte Chemoattractant Protein-1), as also seen in exhaustive endurance exercise [46][93]. The myokine IL-6, a myokine, stimulates other inflammation-limiting cytokines and inhibits IL-1 and TNF production [94]^,^[95]. Thus it may be postulated that the increased inflammatory responses observed during HIIE may be reduced by autocrine adaptive responses [93]. Inflammatory cytokines interact with glucocorticoids and catecholamines[25]. Glucocorticoids suppress most of the production of the three inflammation-promoting cytokines IL-1, IL-6, and TNF, while catecholamines stimulate IL-6, which inhibits IL-1 and TNF and stimulates glucocorticoid secretion promoting the acute-phase response and eventually control of inflammation [96].

### Growth hormone

There is a small amount of data regarding HIIE exercise-induced GH secretion. Significant increases of GH secretion are observed following a single bout of HIIE [97]. A pilot study in young sedentary women compared continuous exercise (cycling for 30 min at 70% VO2max**)** to repeated bouts of sprint exercise (i.e. SIE: 30 s all out sprints with 4.5 min of active recovery) [98]. They found that exercise-induced GH secretion was similar in SIE and continuous exercise [98]. Sprint-type high-intensity efforts generally result in an increase of GH secretion [99]. However, despite an increase in GH, serum concentrations of total IGF-I, free IGF-I, total IGF-II, and IGF-I bioactivity do not change after sprinting, as shown in blood samples collected immediately after, 10 and 30 min post-recovery [100]. Also, lipolysis suppression following nicotinic acid administration was associated with increased GH response to sprint, suggesting the known direct inhibitory role of free fatty acids (FFA) upon GH secretion from the pituitary [101][102][103]. Age is also an important modulator of the GH response to exercise, with younger individuals exhibiting a higher GH increase after sprint exercise [104]. Interestingly, repeated bouts of sprint exercise on the same day result in an attenuation or even disappearance of the exercise-induced increase in GH. Notably, repeated bouts of exercise enhance HPA axis activity and cortisol concentrations, while long-lasting (over 12 h) elevation of glucocorticoids inhibit GH secretion and, subsequently, growth *via* hypothalamic somatostatin secretion [105].

Interestingly, repeated bouts of sprint exercise, in short intervals, on the same day result in an attenuation or even disappearance of the exercise-induced GH increase, though not below resting values, while repeated bouts of exercise enhance HPA axis activity and cortisol concentrations [105][106, 107] [108]. Of note, long treatments with glucocorticoids inhibit GH secretion, probably *via* somatostatin secretion [109].

There is also evidence that speed-endurance training results in a blunted GH response to repeated bouts of sprint exercise, despite an improvement in sprint performance according to a study in healthy non-obese males volunteers who performed sprint interval training for 6-week [110]^,^[97]. Thus, HIIE seems either to reduce or to cause no effect on GH secretory response.

## Endocrine responses of the stress system to a single bout of resistance exercise or to regular resistance exercise

### HPA axis

The response of the HPA axis to resistance exercise depends on the intensity and volume of resistance exercise performed. A single bout of resistance exercise may acutely increase cortisol secretion [111]. The greatest ACTH and cortisol elevations are noticed with resistance protocols involving large muscle mass, with moderate to high loads, high total volume and short rest intervals [112]. Cortisol contributes to muscle remodeling by regulating muscle protein content through muscle protein synthesis inhibition and stimulating protein degradation, likely *via* activation of the ubiquitin proteasome pathway [113]. In addition cortisol influences neuromuscular function and neuronal activity, through various rapid or short-term mechanisms *via* intracellular signaling [114]. Cortisol responses depend on workout design, nutrition (carbohydrate and amino acid administration) [115], genetics, training status and type of resistance training [114]. For example, the type of muscle contraction may modulate HPA axis responses to resistance exercise. There is evidence suggesting that, at similar absolute load, cortisol concentrations increase less over time with concentric (shortening) muscle actions compared to eccentric (lengthening) muscle actions [112]. Also, the type of resistance training may determine whether the HPA axis is stimulated. In the past we have shown that circuit resistance training, which included 10 exercises for different muscle groups performed one after the other in three consecutive “rounds”, separated by 3 min of rest (total of 30 min), resulted in catecholaminergic, but not HPA axis stimulation and a mild inflammatory reaction [116]. This may be due to the fact that circuit training typically involves submaximal loads, while it significantly activates oxygen-dependent metabolism.

HPA axis stimulation by regular resistance exercise is generally characterized by reduced resting cortisol concentrations [117]. The cortisol response to a single bout of resistance exercise is attenuated following a period of resistance training in already trained individuals [118], especially when post-training measurements are made when exercising against the same absolute load [119]. In contrast with the traditional iso-inertial loading (i.e. lifting and lowering the same load), the use of increased load during the eccentric phase (a method called “accentuated eccentric loading”), results in the maintenance of cortisol, testosterone and GH response to a single bout of resistance exercise, which may facilitate ongoing muscle adaptations [118].

### Catecholamines

Circulating catecholamines are important for energy metabolism during resistance exercise [120]. Several studies have shown an increase in epinephrine and norepinephrine concentrations following a single bout of resistance exercise [121]. Plasma epinephrine and norepinephrine concentrations peak towards the end of resistance exercise, especially if the latter is conducted in a “circuit” fashion [116]. The role of circulating catecholamine concentrations may extend further from their typical role as stress hormones. In Ramel et al., “circuit” resistance training (10 exercises against resistances of 75% of maximal strength) was followed by secretion of noradrenaline. Also, secretion of noradrenaline was paralleled by the secretion of ascorbic acid from adrenal gland as well as by the secretion of other antioxidant substances. This parallel increase might indicate a possible direct and/or indirect modulating role of noradrenaline in antioxidant defense, although a confounding effect cannot be excluded [122]. Age, sex and degree of obesity may influence plasma catecholamine responses to intense resistance exercise, but these effects have not yet been well examined [123]. In the past, we have shown, in nine lean and eight obese healthy untrained men who participated in 30 min circuit resistance training, that tissue triacylglycerol lipase activity (TGLA) increased in parallel to the rise of catecholamines [124]. It appears that resistance exercise upregulates adipose tissue lipolysis and enhances energy expenditure in both lean and obese men, with a delayed lipolytic action through TGLA in obese men. Apart from catecholamine increase, it cannot be excluded that this lipolysis increase might be regulated by other molecules (i.e. GH, glucocorticoids) [124]. Up to date, there is minimal evidence for the effects of regular resistance training on catecholamine response.

### Role of cytokines

In resistance exercise, an increase in plasma TNF concentrations, 30 min from the onset of exercise is observed [116]. Muscles are damaged during exercise and there is marked inflammation following resistance training [125]. Inflammatory molecules attract monocytes, macrophages, fibroblasts, and endothelial cells, locally in the damaged muscles, which in turn, produce inflammation-promoting cytokines such as TNF [126]. Increased circulating TNF concentrations were reported during a single bout of resistance exercise in middle-aged women who executed a 60-min resistance exercise program against loads of 50% 1RM [127]. In another study, TNF mRNA measured in muscle biopsy samples increased following resistance exercise [128]. The most plausible source of TNF mRNA is the inflammatory cells residing in the muscle. In a study examining resistance exercise, the observed low cortisol increase was responsible, according to the authors, for the increase in circulating TNF concentrations [129]. Of note, among all inflammation-promoting cytokines cytokines, TNF demonstrates the greatest sensitivity to suppression by cortisol [130].

In contrast to TNF, it is unclear whether other inflammation-promoting cytokines such as IL-1α, IL-1β, and IL-6 are increased in a similar way. In some studies IL-1α, IL-1β, and IL-6 showed no statistically significant changes [116] or only modest increase in IL-6 mRNA after 2 h of intense weight lifting exercise of [131], even though other markers of the muscle damage, such as swelling and soreness of the muscles, were present [132]. Ιn the past, we have shown that IL-6 is a potent stimulus of the HPA axis [41]. The absent or modest IL-6 increase may explain why resistance exercise leads to stimulation of catecholamines rather than the HPA. Interestingly, IL-6 is elevated and may stay at increased concentrations for up to 24–48 h, after heavy eccentric exercise, thus indicating the possible relationship between muscle damage and IL-6 response in this type of training [133].

Resistance exercise-induced inflammation stimulates the secretion of the inflammation-limiting cytokine IL-2, by promoting muscle monocyte differentiation to dendritic cells, which also produce IL-2 [134]. However, other studies have failed to show IL-2 increase following resistance exercise [132]. Concentrations of other inflammation-limiting cytokines such as IL-8 (a chemokine produced by macrophages, epithelial cells and endothelial cells) and IL-10 (an inflammation-limiting cytokine produced primarily by monocytes) do not change following resistance exercise [131].

The response of cytokines to resistance training has not been thoroughly investigated. Some studies reported decreased concentrations of the inflammation-limiting cytokines IL-4 and IL-10, as well as of the inflammation-promoting cytokine interferon γ, in response to repeated bouts of resistance exercise during an 8 week program performed twice per week, in individuals with multiple sclerosis [135]. On the contrary others have failed to corroborate these results in similar patients [136]. In healthy individuals 7 weeks of heavy resistance training resulted in increased IL-6 responses to resistance exercise, coupled with a concomitant increase of IL-1β [119]. However, a muscle biopsy study in young and old men showed that a single bout of resistance exercise (repeated sets of isokinetic knee extension/flexion) was followed by greater IL-6 concentrations in skeletal muscle in the older men, while repetition of this exercise (3 times a week) over 12 weeks led to attenuation of this response [51]. Baseline IL-6 concentrations were greater in elderly than in young men before training. Cytokine responses and oxidative stress biomarkers such as isoprostanes and GSH/GSSG (reduced oxidized glutathione ratio) are correlated to training load and may serve as a tool for overtraining diagnosis [137].

### Growth hormone

Following an intense single bout of resistance exercise, GH concentrations increase markedly, especially when the rest periods between exercises are short, i.e. below 90 s and the total work is large [138]. During resistance exercise, GH concentrations correlate positively with blood lactate concentrations [139]. Notably, endurance exercise elicits a greater GH response than sprint and resistance exercise in young (18–25 years) or middle-aged (40–50 years) men, with responses typically lower in middle-aged, compared with young men [140]. The increase of GH following resistance exercise is transient (15–30 min)[141], followed by IGF-I production in liver and muscles [142]. IGF-I is a critical factor for coordinating muscle growth and repair following resistance exercise [143]^,^[144]. Exercise of any muscle group promotes endocrine adaptive responses with potential benefits for all muscles in the whole body, while exercise-induced increase of IGF-1 concentrations exert neuroprotective effects [145]. However, in West et al. resistance exercise-induced increase of serum anabolic hormones (testosterone, GH, IGF-I) did not improve muscle growth and strength of non-exercising muscles suggesting that muscle growth, hypertrophy and strength may be affected by local mechanisms apart from circulating hormones [146]. The exact role of GH on muscle regeneration and hypertrophy remains to be fully elucidated.

GH stimulation during regular resistance exercise does not alter baseline GH concentrations [147]. The difference between pre- and post- exercise GH concentrations remain similar in regular resistance training protocols of variable duration (3 to 8 weeks) [148].

## Discussion

Although physical activity is becoming part of the lifestyle of children and adults, a large percentage of the population remains sedentary [149]. Physical inactivity exerts deleterious effects to all body systems [10]^,^[150]. Lack of time is put forth as the most important barrier to exercise and thus shorter and more intense types of exercise training are preferred over longer and lower intensity sessions [13]. In the past, we have suggested that exercise represents a stress model [151]. In this review we examined the effects of three different types of exercise (endurance, HIIE, resistance) and their effects upon the stress system-related endocrine responses (namely HPA axis, catecholamines, cytokines, GH, PRL). Since each of these three exercise types imposes different metabolic stress on the organism, we have described their distinct hormonal and cytokine responses, in an attempt to demonstrate the powerful and wide range of effects of exercise on homeostasis and allostasis. This may allow for the appropriate use of exercise types in different target groups during health and disease. It is important to consider which type of exercise and what threshold is suitable for different target groups of exercising people.

A single bout of endurance exercise performed at an intensity higher than 60% VO_2_ max causes IL6 secretion and subsequently intense stimulation of the HPA axis and cortisol secretion. Thus, it represents a stress stimulus which leads to both a local tissue response as well as a potent generalized response of the stress system [152]. Also, it leads to stimulation of the LC/NE system and of GH and PRL release. On the other hand, it seems that stress response to regular endurance exercise consists of moderate HPA axis stimulation in face of increased IL-6 and cortisol baseline concentrations suggesting the existence of adaptive mechanisms of the HPA axis to chronically elevated IL-6 concentrations [6]. In addition, both a single bout and regular endurance exercise lead to similarly increased GH concentrations. Prolactin response to regular endurance exercise needs to be further investigated. Thus, as we have suggested in the past, it appears that stress system stimulation by either a single bout or regular endurance exercise mobilizes adaptive mechanisms as part of the endocrine responses, and it represents a good model for the study of endocrine responses in stress [153].

In healthy individuals a single bout of HIIE stimulates HPA axis, while it seems to acutely decrease cortisol secretion in obese people, who typically present with elevated baseline cortisol concentrations [81]. In addition, a single bout of HIIE leads to increased epinephrine, norepinephrine, IL-6, TNF and GH secretion. On the other hand, regular HIIE results in reduced catecholamine responses suggesting that COMT(Catechol-O-methyltransferase) activity might be influenced by exercise intensity [87]. There is not enough evidence regarding the effect of regular HIIE on HPA axis, cytokines, GH and PRL responses. However, some studies showed a blunted GH response to regular HIIE [110].

A single bout of resistance exercise is characterized by mild HPA axis stimulation depending on exercise intensity and volume, while it increases catecholamine responses and peripheral TNF concentrations. The effect of resistance exercise on IL-6 is still under investigation. However, it seems that IL-6 increases following heavy eccentric exercise, thus indicating the possible relationship between muscle damage and IL-6 increase in this type of training [133]. A single bout of resistance exercise leads to increased GH secretion depending on the exercise intensity, but regular resistance exercise does not alter GH baseline concentrations. regular resistance exercise does not seem to stimulate cortisol secretion even after repeated bouts of resistance exercise [116]. The effects of regular resistance exercise upon catecholamines and cytokine secretion remain unclear.

Understanding different types of exercise and their effects on the endocrine system will help practitioners to provide appropriate exercise prescription to individuals of different training status, age and health level. Endurance exercise and HIIE may prevent over-activation of immune/inflammatory responses in people with autoimmune disorders by increasing cortisol concentrations [154]. On the other hand, resistance exercise is related to lower levels of cortisol concentrations and may be suitable to older individuals particularly those with heart failure or diabetes. Most types of exercise are associated with increased GH concentrations; thus, they may be beneficial for overweight people or for patients with depression, decreased cognitive function, osteoporosis or with low muscle mass. Increased PRL concentrations have been linked with overtraining, but this association requires further investigation.

## Conclusion

This review summarizes the effects of different types of exercise on endocrine function and markers of the immune system (Table 1). A single bout of endurance exercise induces cortisol increase, while regular endurance exercise-induced activation of the HPA axis results to relatively increased basal cortisolemia but the exercise-induced cortisol response is moderate, suggesting the development of HPA axis adaptive mechanisms to the chronically repeated stressor of exercise. Both a single bout and regular endurance exercise induce similar GH peak responses. A single bout of HIIE induces cortisol increase and regular HIIE training lowers basal cortisol concentrations, while catecholamine response is reduced in regular compared with a single bout of HIIE. HPA axis response to resistance exercise depends on the intensity and volume of the exercise. A single boost of resistance exercise is characterized by mild HPA axis stimulation while regular resistance training in elderly results in attenuated inflammatory response and lower resting cytokine concentrations.

More research is needed to further examine their different adaptive responses and the targeted prescription of different types of exercise in healthy and diseased individuals.
